# The Air Quality Health Index and Asthma Morbidity: A Population-Based Study

**DOI:** 10.1289/ehp.1104816

**Published:** 2012-10-10

**Authors:** Teresa To, Shixin Shen, Eshetu G. Atenafu, Jun Guan, Susan McLimont, Brian Stocks, Christopher Licskai

**Affiliations:** 1 Child Health Evaluative Sciences, The Hospital for Sick Children, Toronto, Ontario, Canada; 2 Dalla Lana School of Public Health, University of Toronto, Toronto, Ontario, Canada; 3 Institute for Clinical Evaluative Sciences, Toronto, Ontario, Canada; 4 University Health Network, Toronto, Ontario, Canada; 5 Ontario Lung Association, Toronto, Ontario, Canada; 6 Department of Medicine, University of Western Ontario, London, Ontario, Canada; 7 St. Joseph’s Health Care, London, Ontario, Canada

**Keywords:** air pollution, air quality health index, asthma, health services utilization

## Abstract

Background: Exposure to air pollution has been linked to the exacerbation of respiratory diseases. The Air Quality Health Index (AQHI), developed in Canada, is a new health risk scale for reporting air quality and advising risk reduction actions.

Objective: We used the AQHI to estimate the impact of air quality on asthma morbidity, adjusting for potential confounders.

Methods: Daily air pollutant measures were obtained from 14 regional monitoring stations in Ontario. Daily counts of asthma-attributed hospitalizations, emergency department (ED) visits, and outpatient visits were obtained from a provincial registry of 1.5 million patients with asthma. Poisson regression was used to estimate health services rate ratios (RRs) as a measure of association between the AQHI or individual pollutants and health services use. We adjusted for age, sex, season, year, and region of residence.

Results: The AQHI values were significantly associated with increased use of asthma health services on the same day and on the 2 following days, depending on the specific outcome assessed. A 1-unit increase in the AQHI was associated with a 5.6% increase in asthma outpatient visits (RR = 1.056; 95% CI: 1.053, 1.058) and a 2.1% increase in the rate of hospitalization (RR = 1.021; 95% CI: 1.014, 1.028) on the same day and with a 1.3% increase in the rate of ED visits (RR = 1.013; 95% CI: 1.010, 1.017) after a 2-day lag.

Conclusions: The AQHI values were significantly associated with the use of asthma-related health services. Timely AQHI health risk advisories with integrated risk reduction messages may reduce morbidity associated with air pollution in patients with asthma.

Asthma is a common chronic respiratory disease with a worldwide prevalence ranging from 5 to 18% (Bousquet et al. 2007; Farrar 2005; Masoli et al. 2004) marked by inflammation, bronchial hyperresponsiveness, and airflow limitation. Acute asthma attacks that result in health services use are common (Carlton et al. 2005; Chapman et al. 2001; FitzGerald et al. 2006; Lai et al. 2003; Rabe et al. 2004; Sekerel et al. 2006) and have been associated with a variety of air pollutants (Gilliland 2009; Lin et al. 2005; Stieb et al. 2002, 2009; Weinmayr et al. 2010). Six pollutants are considered in the reporting of air quality in North America using the Air Quality Index (AQI): ground-level ozone (O_3_), fine particulate matter (PM ≤ 2.5 µm in aerodynamic diameter; PM_2.5_), nitrogen dioxide (NO_2_), sulfur dioxide (SO_2_), carbon monoxide (CO), and total reduced sulfur (TRS) compounds. Since 1988, AQI values in Ontario have been established by the Ministry of the Environment to reflect air quality management objectives to protect human health. The AQI is based on the six pollutants noted above and is reported as the value for the single pollutant with the highest AQI (Balluz et al. 2007; Ontario Ministry of the Environment 2012; Shenfeld and Yap 1989). Health Canada and Environment Canada began a collaboration in 2001 to develop a new index named the Air Quality Health Index (AQHI), which was derived based on the combined impact of three pollutants (NO_2_, O_3_, and PM_2.5_) (Environment Canada 2012a). AQHI values are linked to specific risk-reduction health messages designed to educate individuals on the impact of air quality on health, and to advise specific risk reduction actions (Table 1) (Environics Research Group Ltd 2005; Environment Canada 2012b).

**Table 1:** Risk levels and health messages according to AQHI levels (Environment Canada 2012b).

Health risk	AQHI	Health messages		
		At-risk populationa	General population
Low	1–3	Enjoy your usual outdoor activities.	Ideal air quality for outdoor activities.
Moderate	4–6	Consider reducing or rescheduling strenuous activities outdoors if you are experiencing symptoms.	No need to modify your usual outdoor activities unless you experience symptoms such as coughing and throat irritation.
High	7–10	Reduce or reschedule strenuous activities outdoors. Children and the elderly should also take it easy.	Consider reducing or rescheduling strenuous activities outdoors if you experience symptoms such as coughing and throat irritation.
Very high	> 10	Avoid strenuous activities outdoors. Children and the elderly should also avoid outdoor physical exertion.	Reduce or reschedule strenuous activities outdoors, especially if you experience symptoms such as coughing and throat irritation.
aPeople with heart or breathing problems are at greater risk.						

The AQHI has been shown to predict all-cause mortality data in Canada (Stieb et al. 2008), but the AQHI has not been evaluated as a predictor of morbidity, which may be particularly important for conditions such as asthma where mortality is low. In this study, we examined associations between daily values of the AQHI and health services use for asthma, as an indication of the relationship between air quality and asthma morbidity, in the province of Ontario, Canada, from 2003 to 2006.

## Methods

*Data source.* Our study was based in Ontario, Canada’s largest province, which has a multicultural population of > 12 million residents (more than one-third of Canada’s total population). The provincial health system is organized into 14 local health integration networks (LHINs). Ontario has a universal, single-payer health-care system that covers all physician and hospital services, and the personal health information collected for the administration of this system is available in three large databases maintained by the Institute for Clinical Evaluative Sciences (Toronto, Canada). The Ontario Health Insurance Plan Database contains information (including diagnoses) on all fee-for-service billings for physician services rendered in Ontario since 1 July 1991. The Canadian Institute for Health Information Database records the primary and secondary diagnoses for all patients discharged from acute-care hospitals. The Ontario Registered Persons Database includes information on sex, birth date, and residence postal code. We linked these databases together on an individual patient level using an encrypted version of the unique Ontario health insurance number given to all Ontario residents. Such linkage allows for protection of the identities of individual patients while examining their health services use across health administrative databases.

*Study population.* The Ontario Asthma Surveillance Information System (OASIS) Database [maintained by the Institute for Clinical Evaluative Sciences (Toronto, Canada)] is a validated registry of all Ontario residents with asthma and was generated by using the Ontario Health Insurance Plan and Canadian Institute for Health Information health administrative databases described above. To compile the OASIS database, patients with asthma were identified using a previously validated asthma case definition, as described in detail elsewhere and used in previous studies (Gershon et al. 2009; To et al. 2004b, 2006a, 2010). This case definition, which requires at least two physician visits for asthma within 2 consecutive years, or at least one asthma hospitalization ever, yielded 89% sensitivity and 72% specificity in children (0–17 years of age), and 84% sensitivity and 76% specificity in adults (≥ 18 years of age), compared with physician diagnosis documented in medical charts (Gershon et al. 2009; To et al. 2004b, 2006a, 2010). Patients remain in the OASIS database as part of the asthma population until they move out of the province or die, which is consistent with previous evidence indicating that asthma, once diagnosed, may remit but does not resolve (Stern et al. 2008; van Den Toorn et al. 2000). The present study included data from all patients in the OASIS database who had case-defined asthma from 1 January 2003 to 31 December 2006 (To et al. 2004a, 2006b).

This study was approved by the research ethics boards at The Hospital for Sick Children Research Institute and the Institute for Clinical Evaluative Sciences, Toronto, Canada.

*Air quality measures.* Hourly AQHI calculations and air pollutant measures (NO_2_, O_3_, and PM_2.5_) from 1 January 2003 to 31 December 2006 were obtained from the Ontario Ministry of the Environment for 22 monitoring stations across the 14 Ontario LHINs. Air pollutants were measured hourly, 24 hr/day. For LHINs with more than one monitoring station, a mean daily maximum AQHI was calculated using the maximum daily AQHI measured by the monitors within the LHIN, that is, a LHIN-specific daily maximum AQHI was calculated. All patients living within a given LHIN were assigned the same exposure. The same method of exposure assignment was used to determine exposures to the individual pollutants on which the AQHI is based. For descriptive purposes the LHINs were grouped into North, South, Central, East, and West Ontario regions.

*Asthma-related outcomes.* Daily counts of asthma incidence, prevalence, asthma-attributed hospital admissions, emergency department (ED) visits, and outpatient physician claims were identified from OASIS using *International Classification of Diseases, 10th Revision* (ICD-10; World Health Organization 1992) codes (J45, J46). Each day, new asthma cases not previously identified were included (as incidence) and added to the existing asthma cases (prevalence) from that day forward. Count data were arranged by the 14 LHINs of residence and five age groups (0–4, 5–9, 10–19, 20–59, and ≥ 60 years of age). Asthma incidence and prevalence rates were calculated per 1,000 Ontario residents, whereas rates of hospitalizations, ED visits, and outpatient visits were calculated per 1,000 residents with asthma (i.e., patients who were included in the OASIS database).

*Statistical analysis.* For descriptive analysis, we calculated annual mean daily maximum values of air quality measures and annual rates of asthma incidence, prevalence, and health services use for each year and for the study period as a whole (2003–2006). Poisson regression was used to estimate associations between daily AQHI values or individual pollutant measures and daily health service use, including exposures on the same day (D0) and exposures lagged 1 and 2 days (D1 and D2, respectively). All regression models included offset terms for asthma prevalence and included indicator terms to adjust for age (five groups), season, LHIN, and year. Rate ratios (RRs) from the Poisson regression models were used to estimate associations between asthma-attributed health service and a 1-unit increase in the AQHI or an incremental increase in individual air pollutants (10 ppb for NO_2_ and O_3_; 10 μg/m^3^ for PM_2.5_) (Frome 1983). All tests were performed at a 5% significance level. Associations with the individual pollutant components of the AQHI (NO_2_, O_3_, and PM_2.5_) were estimated using Poisson regression models that included all three pollutants. In addition, all models were stratified by age group and by season. Finally, we derived predicted average daily rates of asthma health services use for each level of AQHI with all model covariates at their mean values. Analyses were performed using SAS version 9.2 (SAS Institute Inc., Cary, NC, USA).

## Results

*Air quality measures.* The overall mean daily maximum AQHI was 3.66 ± 1.29, indicating low-to-moderate health risk (Table 2). The highest mean daily maximum AQHI was 3.87 in 2003, and the lowest was 3.34 in 2006. The mean daily maximum AQHI showed yearly fluctuations. Of the five regions, the Central Ontario region, which includes Toronto, had the highest mean daily maximum AQHI [3.94 ± 1.19 (average over all years of the study)], and the North region had the lowest (3.30 ± 1.17; Table 2). Daily maximum AQHI was highest in the summer (4.07 ± 1.43) and lowest in the fall (3.18 ± 1.19).

**Table 2:** Mean measures of AQHI and asthma outcomes by year, age, season, and region.

Covariate	AQHI (mean ± SD)	Annual asthma incidence and prevalence rate^a^		Annual asthma health services use rate^b^		
				Outpatient visits	ED visits	Hospital admissions
		Incidence^c^	Prevalence^d^			
Year
2003	3.87 ± 1.37	7.11	122.60	622.90	41.71	5.41
2004	3.64 ± 1.18	6.84	124.80	577.20	38.95	5.11
2005	3.83 ± 1.40	7.03	128.20	563.80	39.10	5.38
2006	3.34 ± 1.12	6.65	131.20	524.00	35.22	4.14
2003–2006	3.66 ± 1.29	6.91	126.70	572.00	38.75	5.01
Age group
0–4	NA	41.72	114.86	1759.06	174.96	42.08
5–9	NA	13.54	217.56	622.64	44.34	6.63
10–19	NA	5.67	224.10	315.00	23.37	1.67
20–59	NA	3.90	100.42	526.68	34.56	2.74
≥ 60	NA	4.88	110.17	693.54	27.28	3.84
Season
Spring (Mar–May)	3.95 ± 1.17	7.20	126.08	591.08	40.42	5.20
Summer (Jun–Aug)	4.07 ± 1.43	5.47	126.88	486.52	29.71	3.30
Fall (Sep–Nov)	3.18 ± 1.19	7.59	127.38	628.57	45.92	6.67
Winter (Dec–Feb)	3.45 ± 1.14	7.38	126.51	581.20	38.80	4.85
Region
North	3.30 ± 1.17	5.81	121.69	534.71	56.16	6.92
South	3.74 ± 1.43	5.70	113.82	514.78	37.43	6.10
Central	3.94 ± 1.19	7.91	130.10	622.76	29.70	4.59
East	3.47 ± 1.14	7.21	137.80	561.24	43.75	4.13
West	3.77 ± 1.14	5.74	114.45	529.24	45.73	5.77
NA, not applicable. Data stratified by age group, season, and region are based on data averaged from 2003–2006. aPer 1,000 individuals. bPer 1,000 residents with asthma (population includes all Ontario residents in the OASIS database). cNumber of new cases identified each day not known prior to that day. dSum of current and new cases.												

*Health services use.* The mean annual asthma incidence and prevalence rates per 1,000 Ontario residents during 2003–2006 were 6.9 and 126.7, respectively (Table 2). The annual incidence of asthma fluctuated between a low of 6.7 in 2006 and a high of 7.1/1,000 Ontario residents in 2003. The overall prevalence of asthma increased by 7.0% from 2003 to 2006 (Table 2). The annual mean rates of asthma outpatient visits, ED visits, and hospitalizations over the entire study period per 1,000 residents with asthma were 572.0, 38.8, and 5.0, respectively. All asthma health services outcomes were higher in 2003 than in 2006, although outpatient visits were the only outcome that decreased monotonically over time.

The Central Ontario region had the highest annual mean rate of outpatient visits per 1,000 residents with asthma (622.8), and the North region had the highest mean rate of ED visits (56.2) (Table 2). Annual rates for use of all three asthma health services were highest among 0–4 year olds and lowest among 10–19 year olds, and were highest in the fall and lowest in the summer.

*Adjusted asthma health services RRs.* Daily maximum AQHI was associated with a positive, significant increase in the use of each asthma health service evaluated during at least one lag period (Table 3). The adjusted asthma outpatient visit rate ratio was highest for AQHI on the same day (D0 RR = 1.056; 95% CI: 1.053, 1.058) indicating that a unit increase in the AQHI was associated with an estimated 5.6% increase in asthma outpatient visits. However, there was a significant negative association between asthma outpatient visits and the AQHI 2 days before the visit (D2 RR = 0.983; 95% CI: 0.981, 0.986). The asthma hospitalization rate ratio also was highest for AQHI on the same day and the previous day (both D0 and D1 RR = 1.021; 95% CI: 1.014, 1.028), suggesting a 2.1% increase in hospital admissions attributed to asthma for each unit increase in the AQHI. The RR for asthma ED visits was highest for AQHI 2 days before the visit (D2 RR = 1.013; 95% CI: 1.010, 1.017), suggesting a 1.3% increase in asthma ED visits per unit increase in AQHI.

**Table 3:** RRs (95% CIs) for asthma health outcomes in association with a 1-unit increase in the AQHI.

Lag	Outpatient visits		ED visits		Hospital admissions	
D0
AQHI	1.056	(1.053, 1.058)	1.003	(0.999, 1.007)	1.021	(1.014, 1.028)
NO2	1.117	(1.114, 1.120)	0.976	(0.971, 0.980)	1.025	(1.017, 1.034)
O3	0.979	(0.976, 0.981)	1.008	(1.004, 1.012)	1.017	(1.009, 1.024)
PM2.5	0.982	(0.978, 0.985)	1.028	(1.022, 1.035)	0.997	(0.986, 1.007)
D1
AQHI	1.019	(1.016, 1.021)	1.005	(1.001, 1.009)	1.021	(1.014, 1.028)
NO2	1.022	(1.020, 1.025)	0.976	(0.972, 0.981)	1.011	(1.003, 1.018)
O3	1.018	(1.015, 1.020)	1.014	(1.009, 1.018)	1.031	(1.023, 1.039)
PM2.5	0.990	(0.986, 0.993)	1.022	(1.016, 1.028)	1.002	(0.991, 1.012)
D2
AQHI	0.983	(0.981, 0.986)	1.013	(1.010, 1.017)	1.008	(1.001, 1.015)
NO2	0.959	(0.956, 0.962)	0.994	(0.990, 0.999)	0.991	(0.983, 0.999)
O3	1.016	(1.014, 1.019)	1.010	(1.006, 1.014)	1.043	(1.036, 1.051)
PM2.5	1.006	(1.002, 1.009)	1.017	(1.011, 1.023)	0.992	(0.981, 1.002)

Results from the multipollutant Poisson regression model adjusted for covariates are also shown in Table 3. The highest NO_2_-specific RR was found on D0 for asthma outpatient visits (RR = 1.117; 95% CI: 1.114, 1.120), suggesting a nearly 12% increase in outpatient claims per 10 unit increase in NO_2_. The highest O_3_-specific RR was found on D2 for hospitalizations (RR = 1.043; 95% CI: 1.036, 1.051). The highest PM_2.5_-specific RR was observed on D0 for ED visits (RR = 1.028; 95% CI: 1.022, 1.035).

**Figure 1:**
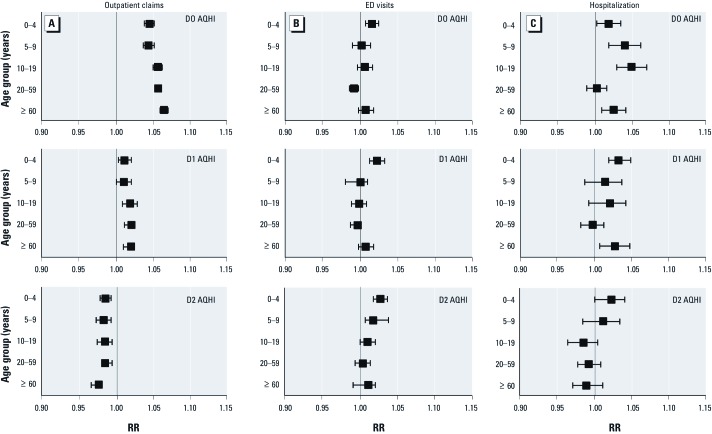
RRs (95% CIs) for asthma health services by AQHI and lags stratified by age group. Outpatient claims (*A*), ED visits (*B*), and hospitalization (*C*) for AQHI on D0 (top), D1 (center), and D2 (bottom). All health services RRs were derived from multivariable poisson regression models adjusted for season, region, and year. The AQHI-specific RRs were per unit increase in AQHI.

**Figure 2:**
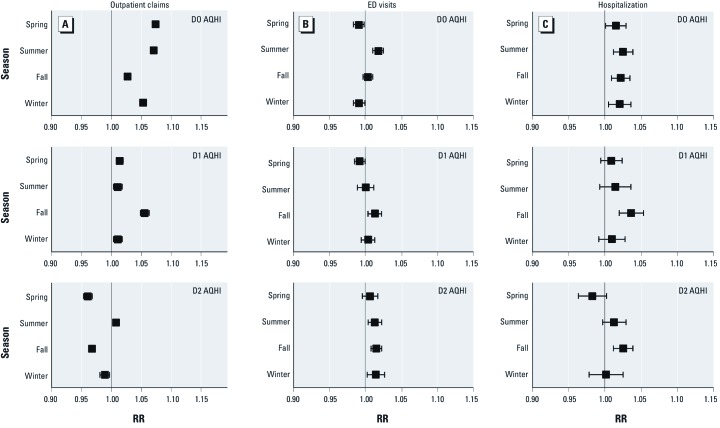
RRs (95% CIs) for asthma health services by AQHI and lags stratified by season. Outpatient claims (*A*), ED visits (*B*), and hospitalization (*C*) for AQHI on D0 (top), D1 (center), and D2 (bottom). All health services RRs were derived from multivariable poisson regression models adjusted for age, region, and year. The AQHI-specific RRs were per unit increase in AQHI.

Figure 1 shows the results of the Poisson regression models stratified by age group. The youngest (0–4 years of age) and the oldest age groups (≥ 60 years of age) had the highest RRs for asthma ED visits on D2 and hospitalization on D1. The oldest age group had the highest RR for asthma outpatient claims on D0. Figure 2 shows results stratified by season. Although the RRs showed no difference in asthma ED visits or hospitalization by seasons, RRs for D0 were higher in the spring and summer for asthma outpatient claims.

NO_2_ was associated with higher asthma outpatient visits and hospitalizations, particularly in the summer; O_3_ had the highest association with outpatient claims in the spring and summer, whereas PM_2.5_ had the highest associations with ED visits and outpatient claims in the winter (Table 3). [For mean air pollutant measures by year, season, and region in Ontario, see Supplemental Material, Table S1 (http://dx.doi.org/10.1289/ehp.1104816).] In general, higher associations were observed in the younger age groups.

*Predicted average daily rate of asthma health services use.* Predicted average daily rates of asthma health services use per unit increase in AQHI at D0 in total and by age group were calculated from the adjusted Poisson regression models. The increase in predicted daily rates of asthma health services use per unit increase in AQHI was highest in the very young and the oldest populations. Table 4 shows predicted daily rates and the expected counts of asthma health services use by AQHI values as applied to an asthma-prevalent population with average values of model covariates. About 1.5 million persons living with asthma in Ontario during the study period based on the provincial population of 12 million and asthma prevalence of 12.6%. The predicted daily rates per 1,000 residents with asthma on days when the AQHI = 3 (indicating low health risk) were 1.498 for outpatient asthma claims, 0.106 for asthma ED visits, and 0.013 for asthma hospitalizations, which we estimate would result in nearly 2,278 outpatient visits, 160 ED visits, and 20 hospital admissions attributed to asthma (Table 4). If the AQHI = 10 (high health risk), these daily expected counts would increase to 3,330, 164, and 24, representing increases of 46%, 2%, and 16% relative to counts on days when AQHI = 3, respectively. As these are daily expected counts calculated from daily rates, the absolute increase in health care burden could be large if more days in a year have higher AQHI measures.

**Table 4:** Predicted daily average rates and daily counts for use of asthma health services according to AQHI levels.

	AQHI = 3 (low health risk)		AQHI = 6 (moderate health risk)			AQHI = 10 (high health risk)		
Asthma Morbidity measures	Predicted rate^a^	Expected number^b^	Predicted rate	Expected number	Percent difference^c^	Predicted rate	Expected number	Percent difference
Outpatient visits	1.498	2,278	1.763	2,681	17.7	2.190	3,330	46.2
ED visits	0.106	160	0.106	162	0.8	0.108	164	2.0
Hospital admissions	0.013	20	0.014	22	6.4	0.016	24	15.7
aPredicted daily average rates were obtained from the adjusted Poisson regression models with age, season, region, and year held at their mean values. bExpected counts were calculated by multiplying the predicted rates to the average asthma prevalence (in the example above, we used the Ontario 1.5 million asthma prevalence population for illustration). cPercent difference compared to AQHI = 3.																

## Discussion

This study extends our understanding of the deleterious health effects of air pollutants by associating asthma morbidity directly with a simple population-based air quality health risk scale. Our results suggest that an increase in the daily maximum AQHI is associated with an increase in asthma health services use. Associations are evident on the day of exposure and for exposure 1 and 2 days before the outcome. The AQHI, as well as individual pollutants, demonstrated associations with health services use.

Our findings are supported by previous studies of individual pollutants and the multivariable AQHI scale (Table 5). According to a study of 12 Canadian cities that included data for nearly two decades, each unit increase in the AQHI was associated with a 1.2% increase in mortality (Stieb et al. 2008). A comprehensive, systematic synthesis of 109 daily time–series studies suggested that acute exposures to air pollutants such as NO_2_, O_3_, and PM_10_ (PM ≤ 10 µm in aerodynamic diameter; thoracic PM) contribute to all-cause mortality, with NO_2_ and PM_10_ showing stronger associations with respiratory mortality (Stieb et al. 2002). Furthermore, a study of 11 Canadian cities from 1980 to 1991 found significant associations between NO_2_ and O_3_ and non-accidental mortality (Burnett et al. 1998).

**Table 5:** Summary of studies examining the association between air quality measures and asthma.

Reference	Data collection period	Location	Study population	Sample size (n or no. studies)	Outcomes	Air quality measures	Findings
Stieb et al. 2008	1981–2000	12 Canadian cities	All ages	NA	Overall mortality	AQHI, SO2, NO2, O3, CO, PM10, PM2.5	Each unit increase in AQHI was associated with an increase of 1.2% in mortality
Stieb et al. 2002	1985–2000	Worldwide	All ages	109 studies	All-cause, respiratory mortality	SO2, NO2, O3, CO, PM10	Acute air pollution exposure was significantly associated with mortality; stronger associations with respiratory mortality for all pollutants except O3
Burnett et al. 1998	1980–1991	11 Canadian cities	All ages	816,991	Mortality of nonaccidental causes	SO2, NO2, O3, CO	All pollutants were significantly associated with mortality; NO2 had the strongest association
Weinmayr et al. 2010	1990–2008	Europe, USA, other	≤ 18 years	36 studies	LRS, cough, PEF of children with asthma	NO2, PM10	PM10 was significantly associated with asthma symptom episode; NO2 was significantly associated with asthma symptoms in overall analysis only considering all possible lags
Stieb et al. 2009	1992–2003	7 Canadian cities	All ages	83,563 (asthma); 125,145 (respiratory)	ED visits for asthma and respiratory infection	SO2, NO2, O3, CO, PM10, PM2.5	Ozone was associated with visits for respiratory conditions; PM2.5 and PM10 were associated with asthma visits in warm season
Lin et al. 2005	1998–2001	Toronto, Canada	≤ 14 years	6,782	Hospitalization for respiratory infection	SO2, NO2, O3, CO, PM10, PM2.5, PM10–2.5	All PM fractions and NO2 were significantly associated with hospital admissions for respiratory infections
Current study 2012	2003–2006	Province of Ontario, Canada	All ages	1.5 million (asthma)	Outpatient, ED visits	AQHI, NO2, O3, PM2.5	AQHI was significantly associated with asthma morbidity on the current day and 1–2 days prior
Abbreviations: LRS, lower respiratory symptoms; NA, not available; PEF, peak expiratory flow; PM10–2.5, PM, with an aerodynamic diameter between 2.5 and 10 µm, coarse PM .											

While AQHI has been associated with mortality, its association with morbidity outcomes has not been fully assessed. Several recent studies have reported associations between individual air pollutants and adverse health outcomes. According to a systematic review of 36 studies, PM_10_ and potentially NO_2_ were significantly associated with the occurrence of asthma symptom episodes among patients ≤ 18 years of age (Weinmayr et al. 2010). A time–series analysis based on nearly 400,000 ED visits at 14 hospitals in seven Canadian cities during the 1990s through the early 2000s concluded that daily average concentrations of O_3_ exhibited the most consistent associations with ED visits for respiratory conditions, and that PM_10_ and PM_2.5_ were strongly associated with visits for asthma during the warm season (Stieb et al. 2009). Furthermore, a 4-year study found associations between hospitalization for respiratory infections in children ≤ 14 years of age in Toronto and relatively low levels of ambient particulate matter and gaseous pollutants, especially PM_10–2.5_ (PM with an aerodynamic diameter between 2.5 and 10 µm) and NO_2_ (Lin et al. 2005).

Although our study is not a formal validation study of AQHI morbidity outcomes, it is the first to use a large body of population-based data to evaluate associations between AQHI and asthma-related morbidity. We used asthma as an index disease because it is very common and is the fastest-growing chronic disease in North America, and because air pollutants have been associated with asthma symptoms and exacerbations. Recent studies have suggested that other chronic diseases may also be aggravated by air pollution, including chronic obstructive pulmonary disease, heart disease (including heart attack and stroke), and diabetes (Andersen et al. 2012; Hoffmann et al. 2012; Ko and Hui 2012; Lavigne et al. 2012; Wellenius et al. 2012). Our study supports the utility of AQHI as an exposure metric for studies of the impact of ambient air pollution on health outcomes, and our approach may serve as a prototype for studies of the impact of air quality on other chronic diseases.

The use of large health administrative and environmental databases helped ensure the comprehensiveness, representativeness, and generalizability of our findings while minimizing selection bias, but there are some limitations. We used a large population-based database from Canada, potentially limiting the generalizability of our findings to other populations. The AQHI, a recent Canadian innovation, is an index of air quality that is focused on health risk and on the communication of that risk to the general public; however, at this time the AQHI is not used outside of Canada. Although our estimates were adjusted for several confounding factors, we could not account for other potential confounders such as smoking, housing conditions, indoor air quality, and ethnicity. Because all persons residing within a given region were assigned the same level of exposure without formally accounting for variations within the region, there is the potential for misclassifying exposure. In addition, health administrative data may underestimate morbidities associated with asthma and misdiagnosis was possible. However, we attempted to reduce the misclassification of outcomes by using a validated and highly specific case definition of asthma.

The multivariable analyses in our study were conducted using fixed-effect Poisson regression models that adjusted for confounders including region and year. Because our study used data from 2003 to 2006 obtained for various regions in Ontario, there may be some degree of spatial autocorrelation as well as time dependency in the data for which we have not fully accounted. Methods used by others that take into account spatial autocorrelation include complex regression approaches such as Poisson regressions with distance-based agglomeration-specific spatial random effects and Poisson regressions with neighborhood-based agglomeration-specific spatial random effects (Mohebbi et al. 2011). According to simulation results reported by Mohebbi et al. (2011), ignoring spatial autocorrelation may potentially overstate the degrees of freedom in the data and consequently underestimate standard errors. Even though this error would not affect rate ratio estimates, it is likely that we have overstated their statistical significance. Although it would be desirable to account for residual spatial correlation in analyses, it is challenging to specify the correct correlation structure and apply appropriate spatial smoothing. However, a more sophisticated temporal and spatial analysis could be considered in the future to account for potential autocorrelation and time dependency of the data.

The AQHI is designed to help persons make decisions to protect their health by limiting short-term exposure to air pollution and adjusting their activity when air pollution levels are high. In our study, rate ratios were estimated assuming constant linear associations per unit increase of AQHI. Future studies should examine specific AQHI cut points in relation to the levels of severity of health risks.

The National Illness Cost of Air Pollution (ICAP) study conducted by the Canadian Medical Association in 2008 suggested that respiratory illness associated with exposure to air pollution accounted for a significant burden to the health care system and productivity loss (Canadian Medical Association 2008). Our study suggests a statistically significant increase in asthma health services use per unit increase in AQHI also exists. The AQHI health messages providing recommendations on how to adjust outdoor activity levels in accordance with AQHI levels may play an important role in informing persons about health risks and air pollution and may contribute to reducing unnecessary health care use due to adverse health outcomes attributable to exposure to air pollution.

## Conclusion

Our study was the first to use population data to study associations between asthma morbidity and the AQHI. Daily rates of asthma health services use predicted on the basis of our estimates may be useful for health care resource allocation and planning and may serve as a guide for the timing of asthma education and management interventions and air quality risk reduction campaigns.

Our findings support the use of the AQHI as a chronic disease morbidity index. As an air quality health risk advisory tool, the composite AQHI reflects the combined effects of ambient air pollutant exposures relevant to patients with asthma. Furthermore, the AQHI was developed as a communication tool that includes simple risk-reduction advice, permitting practical implementation as an asthma trigger avoidance management strategy. The AQHI may be useful for forecasting asthma morbidity associated with outdoor air pollution, and education about the AQHI may help reduce health services use by patients living with asthma.

## Supplemental Material

(70 KB) PDF
