# Supplementary material for: The Air Quality Health Index and Asthma Morbidity: A Population-Based Study
Source: Environ Health Perspect. 2012 Oct 10;121(1):46–52. doi: 10.1289/ehp.1104816 (PMC3546347; doi:10.1289/ehp.1104816)
Supplement: (70 KB) PDF [file ehp.1104816.s001.pdf]

Supplemental Material

The Air Quality Health Index and Asthma Morbidity: A Population-Based Study

Teresa To, Shixin Shen, Eshetu G. Atenafu, Jun Guan, Susan McLimont, Brian Stocks, Christopher Licskai

Supplemental Material Table S1. Mean air pollutant measures by year, season and regions in Ontario

Covariates	NO_2 (ppb)	O_3 (ppb)	PM2·5 (μg/m3)
Year			
2003	28·55 ± 13·51	40·03 ± 15·21	14·62 ± 9·78
2004	26·72 ± 12·16	37·12 ± 12·74	13·61 ± 9·74
2005	27·07 ± 13·49	40·93 ± 16·18	14·48 ± 11·19
2006	22·12 ± 11·87	38·20 ± 14·28	12·43 ± 8·50
2003 to 2006	26·05 ± 12·99	39·07 ± 14·73	13·78 ± 9·88
Correlation Coefficients[a]			
NO_2 and O_3	-0.1146 (p=0.0004)		
NO_2 and $PM_{2.5}$	0.0969 (p=0.0026)		
O_3 and $PM_{2.5}$	0.6500 (p<0.0001)		
Season[b]			
Spring (Mar-May)	28·97 ± 10·86	44·33 ± 9·86	12·49 ± 8·23
Summer (Jun-Aug)	21·71 ± 10·86	49·61 ± 16·26	18·55 ± 12·06
Fall (Sep-Nov)	23·69 ± 10·86	32·32 ± 13·24	13·21 ± 9·83
Winter (Dec-Feb)	29·88 ± 13·23	29·76 ± 7·68	10·86 ± 6·87
Region[b]			
North	23·34 ± 13·60	38·29 ± 11·90	11·16 ± 8·13
South	24·37 ± 11·59	39·65 ± 14·98	14·85 ± 10·26
Central	30·64 ± 13·24	39·50 ± 15·84	14·94 ± 10·66
East	23·42 ± 12·56	38·17 ± 14·72	13·65 ± 9·81
West	25·58 ± 10·83	40·13 ± 15·87	14·49 ± 9·60

[a] Correlation coefficients were based on Pearson Correlation analysis; p stands for p-value.

[b] Data stratified by season and region are based on data averaged from 2003-2006.
